# Taxonomic novelties in *Columnea* (Gesneriaceae) from Peru: Two new species from the Bosque de Protección Pui Pui

**DOI:** 10.3897/phytokeys.275.187262

**Published:** 2026-05-19

**Authors:** Rocío del Pilar Rojas Gonzáles, Rodolfo Vásquez Martínez, Elmes Pinche Shareva

**Affiliations:** 1 Herbario Selva Central Oxapampa – HOXA, Jardín Botánico de Missouri, Prolongación Bolognesi Mz. E–6, Oxapampa, Pasco, Peru Herbario Selva Central Oxapampa – HOXA, Jardín Botánico de Missouri Oxapampa Peru

**Keywords:** *
Columnea
*, distribution, endemics, Neotropics, Peru, taxonomy

## Abstract

*Columnea puipuiense* R.Rojas & Vásquez, **sp. nov**., and *Columnea valenzuelai*, R.Rojas & Vásquez, **sp. nov**., two new species of Gesneriaceae endemic to the Bosque de Protección Pui Pui, central Peru, are described and illustrated. Their morphological affinities with related species of the genus *Columnea* are discussed, and complete descriptions are provided, including diagnostic characters, ecological information, and geographic distribution. With the recent discovery of *Columnea yanachagaensis* R.Rojas, A.Monteagudo & J.Flores and *C. cesarii* R.Rojas, Vásquez & L. Valenz, along with the discovery of *Columnea puipuiense* and *Columnea valenzuelai*, the number of *Columnea* species recorded for Peru increases to 36. These findings raise the documented diversity of Peruvian Gesneriaceae to 227 species.

## Abstract (es)

Se describen e ilustran *Columnea puipuiense* R.Rojas & Vásquez, **sp. nov**., y *Columnea valenzuelai* R.Rojas & Vásquez, **sp. nov**., dos nuevas especies de Gesneriaceae endémicas del Bosque de Protección Pui Pui, selva central del Perú. Se comparan sus afinidades morfológicas con especies relacionadas del género *Columnea*, y se presenta una descripción completa que incluye caracteres diagnósticos, información ecológica y datos sobre su distribución geográfica. Con los recientes descubrimientos de *Columnea yanachagaensis* R.Rojas, A.Monteagudo & J.Flores y *Columnea cesarii* R.Rojas, Vásquez & L. Valenz, junto con *Columnea puipuiense* y *Columnea valenzuelai*, el número de especies de *Columnea* registradas para el Perú se incrementa a 36. Estos hallazgos elevan la diversidad documentada de Gesneriaceae peruanas a 227 especies.

## Introduction

The Bosque de Protección Pui Pui, known as the “spring of the Pui Pui mountain range,” is a protected natural area classified for direct use, where conservation objectives are explicitly combined with the sustainable use of natural resources by local populations. The area covers approximately 60,000 hectares and contains about 99 lagoons, which constitute aquatic ecosystems that serve as habitats for aquatic and semi-aquatic flora. The Bosque de Protección Pui Pui encompasses the following ecoregions: the Peruvian Yungas, which includes montane forest, cloud forest, and upper montane rainforest, and the Humid Puna of the Central Andes, which corresponds to high-Andean grasslands (SERNANP 2019). Since October 2020, the Bosque de Protección Pui Pui has been part of the Biosphere Reserve Bosque de Neblina-Selva Central (SERNANP 2022).

Since 2014, our team of botanists has conducted systematic explorations within the protected natural area, leading to the discovery of several plant species new to science, including *Brachionidium puipuiensis* L. Valenz., of the Orchidaceae (Valenzuela Gamarra 2017), *Polylepis rodolfo-vasquezii* L. Valenz. & Villalba (Valenzuela Gamarra and Villalba Valdivia 2015), and *Polylepis rocio-rojasiae* L. Valenz. & Villalba of the Rosaceae (Valenzuela Gamarra and Villalba Valdivia 2024). It is noteworthy that the area remains poorly documented in terms of its vegetation; therefore, our research has focused on repeated botanical surveys aimed at achieving a more comprehensive understanding of its flora. As a result of these efforts, two new species of *Columnea* have been identified, contributing to the current knowledge of the vascular flora of the study area.

*Columnea* is distinguished from its congeners by the presence of indehiscent fleshy berries (Schulte et al. 2014), except for *C. dielsii* Mansf., *C. cesarii* R.Rojas, Vásquez & L.Valenz. and *C. yanachagaensis* R.Rojas, A.Monteagudo & J.Flores, which have dehiscent fleshy capsules (Clark 2020; Rojas Gonzáles et al. 2025). Most species of *Columnea* are epiphytic, although some occur terrestrially or as facultative epiphytes. The genus exhibits a broad range of growth habits, from epiphytes with dorsiventral shoots to pendent vines with elongate shoots (Clark et al. 2025). *Columnea* is the most diverse genus within the subfamily with more than 220 recognized species (Clark et al. 2020; GRC 2024; POWO 2025) distributed throughout the New World tropics, from southern Mexico to southern Peru and Bolivia, Brazil, the Guianas, and the Caribbean (Skog 1979; Möller and Clark 2013). The genus *Columnea* ranges from lowland rain forests to subpáramo forests (Smith 1994), and its greatest diversity is concentrated in the northern Andes of Colombia and Ecuador, mainly in Andean cloud forests up to 4000 m in elevation (Tobar et al. 2022). In Peru, the genus is mainly restricted to the eastern Andean slopes, where it inhabits humid montane and cloud forests, with peak diversity at mid-elevations and a markedly lower representation in lowland Amazonian forests (Kvist et al. 2005).

This article describes *Columnea puipuiense* R.Rojas & Vásquez and *Columnea valenzuelai* R.Rojas & Vásquez (Figs 2, 3), endemic to the central rainforest of Peru. With this discovery, the number of known species of Peruvian *Columnea* increases to 36, and the number of species in the Gesneriaceae family in Peru to 227 (Rojas Gonzáles 2020; Rojas Gonzáles et al. 2025). This study contributes to the understanding of the diversity and endemism present in Andean Gesnerioideae and highlights the importance of continuing to promote botanical exploration in the country’s poorly studied montane ecosystems, especially within protected natural areas that serve as refuges for many plant species still unknown to science.

## Materials and methods

**Figure 1.**
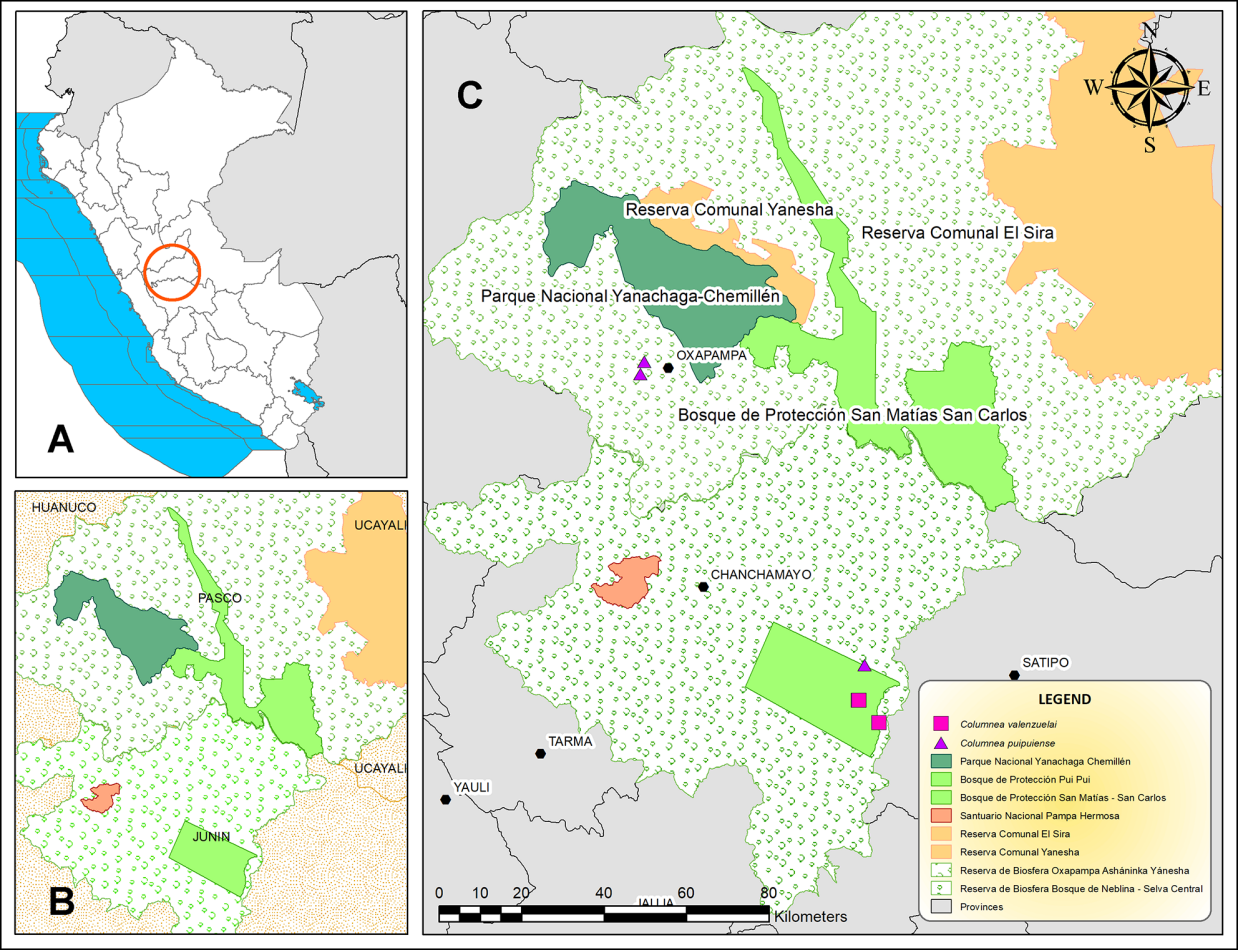
Distribution map of *Columnea puipuiense* and *Columnea valenzuelai*. **A**. Map of Peru and its Departments; **B**. Study area showing protected natural areas in the Central Peruvian rainforest; **C**. Collection locations.

This study was based on morphological analyses conducted using a dissecting stereomicroscope, along with a review of specialized literature. Descriptions were prepared through examination of herbarium specimens deposited at the Selva Central Oxapampa Herbarium (**HOXA**), with the acronym following Thiers (2025). A complete set of specimens was deposited at HOXA, and duplicates, when available, were distributed to the herbaria MO, MOLF, and USM. Species identification was based on a combination of morphological characters. Descriptive terminology followed Smith (1994) and Kvist et al. (1993). Floral descriptions and measurements were obtained from material preserved in 50–70% ethanol. Information on the coloration of vegetative and floral structures was derived from field observations and notes on herbarium labels. The two new species of *Columnea* are distinguished by a unique combination of morphological characters. The geographic distribution map (Fig. 1) was generated using ArcGIS version 10.3 (ESRI 2014). Conservation status was assessed following the guidelines of the International Union for Conservation of Nature. The extent of occurrence (**EOO**) and area of occupancy (**AOO**) were calculated using GeoCAT (Bachman et al. 2011) with a default cell size of 2 km^2^, as recommended by the IUCN (2024).

## Taxonomic treatment

### Columnea puipuiense

Taxon classification

Plantae

LamialesGesneriaceae

R.Rojas & Vásquez sp. nov.

5E12A7BD-32CB-522A-B584-9740CF906171

urn:lsid:ipni.org:names:77380394-1

Fig. 2

#### Type.

Peru • Junín: Prov. Chanchamayo, Dist. Pichanaki, Bosque de Protección Pui Pui, bosque primario, 11°13'33.935"S, 74°58'05.892"W, 1968–2032 m, 8–19 Aug. 2025 (fl.), *R. Rojas, R. Vásquez & J. Flores 12889* (holotype: HOXA [HOXA094631]; Isotypes: MO!, MOL-F!, USM!).

**Figure 2.**
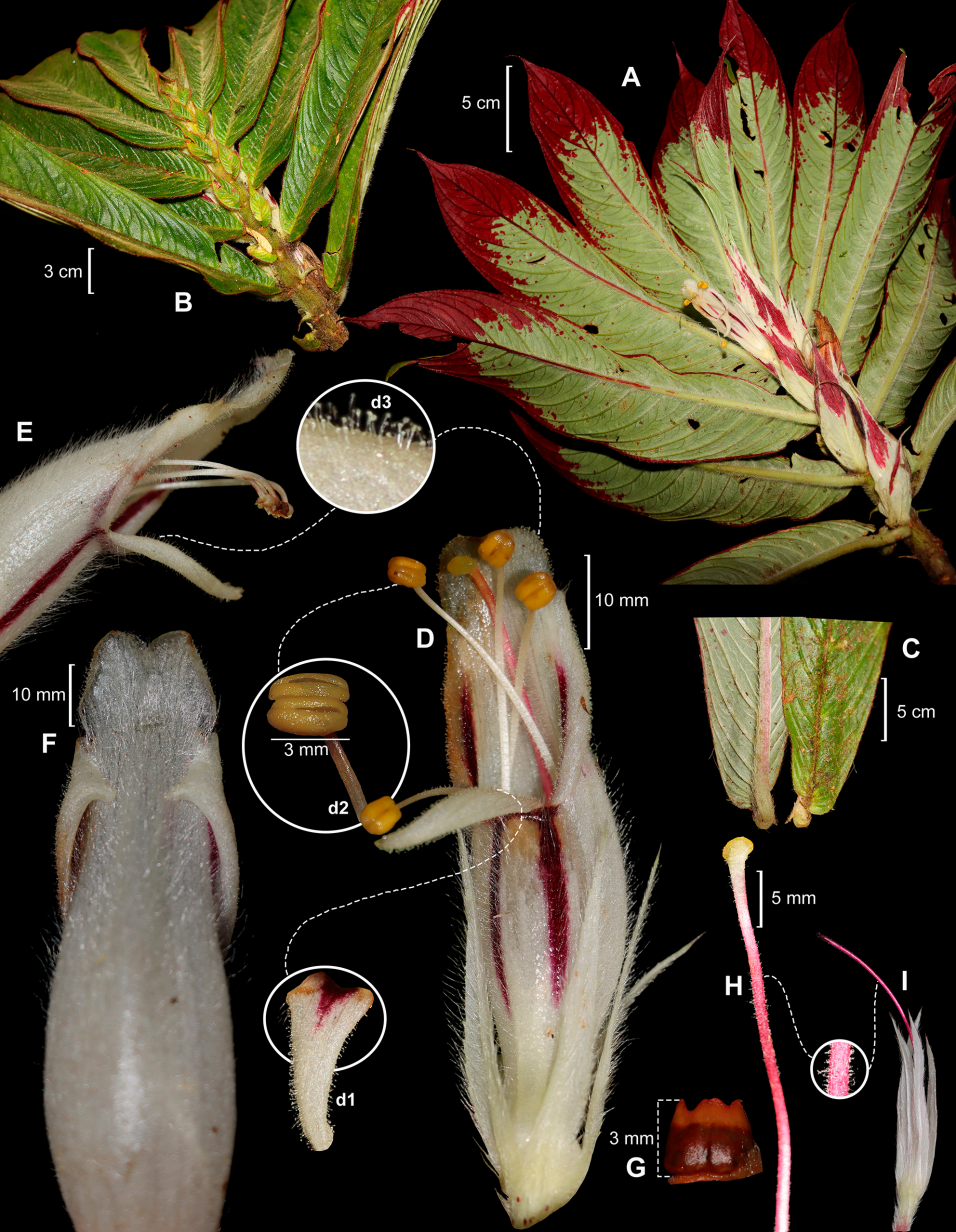
*Columnea puipuiense* R.Rojas & Vásquez. **A**. Habit, showing abaxial leaf surface, bracts, and flower; **B**. Branchlet showing adaxial leaf surface; **C**. Base of leaf blade; **D**. Corolla: lower corolla lobe with a dark maroon-purple basal blotch (**d1**), anthers (**d2**), glandular trichomes (**d3**); **E**. Corolla, lateral view; **F**. Corolla, dorsal view, showing reflexed lateral lobes; **G**. Nectary; **H**. Style and stigma; **I**. Calyx showing style (**A–I** from R. Rojas et al. 12889). Photos by Rocío Rojas. Plate prepared by Rocío Rojas.

#### Diagnosis.

*Columnea puipuiense* shares morphological affinities with *Columnea albiflora* L.P.Kvist & L.E.Skog, *Columnea tessmannii* Mansf. and *Columnea medicinalis* (Wiehler) L.E.Skog & L.P.Kvist; however, it is distinguished by an olive-green, distinctly discolorous abaxial leaf surface with maroon-red coloration at the apex and margins (vs. usually uniformly colored, pale yellowish-green, with reddish primary and secondary veins in *C. albiflora*; generally violet to purple in *C. tessmannii*), a white to cream-white corolla (vs. yellow in *C. tessmannii*; white, cream, or yellow with orange lobes in *C. medicinalis*), and a sericeous-villous indumentum composed of long, hyaline, uniseriate, eglandular trichomes distributed along the entire length (vs. sericeous in *C. albiflora*; sericeous-villous, mostly in the upper portion, in *C. tessmannii*). In addition, it has reflexed lateral corolla lobes (vs. spreading in *C. albiflora*, *C. tessmannii*, and *C. medicinalis*) and a narrowly ovate-triangular, patent, lobe (vs. ovate and reflexed in *C. albiflora*; ovate-lanceolate and reflexed in *C. tessmannii*; lanceolate and reflexed in *C. medicinalis*).

#### Description.

Shrubs, subwoody, epiphytic, perennial; stems erect, ascending, or spreading, to 2 m long, terete in cross-section, 6.5–9.0 mm in diam., densely pilose to villous, especially in young portions; internodes 1.5–3.0 cm long. ***Leaves*** opposite, strongly anisophyllous in each pair, subsessile or with petioles < 10 mm long; larger blade elliptic to lanceolate-elliptic, sometimes tending toward oblanceolate, 16–29(–30) × 3.6–6.5(–7) cm; apex acuminate to long-acuminate; base attenuate to slightly rounded, oblique; adaxial surface green, rugose, appressed-pilose to hirsute; abaxial leaf surface olive green, distinctly discolorous, with maroon-red coloration at the apex and margins, and additional maroon maculation; midrib prominent; secondary veins 13–14(–16) pairs, arcuate, well defined; margin subentire to weakly serrulate, mainly toward the apex; marginal vesture composed of reddish-purple to transparent uniseriate trichomes, occasionally glandular. ***Petiole*** 0.4–0.8(–1.0) cm long, greenish to yellowish, densely pilose to villous, with transparent uniseriate trichome. ***Inflorescence*** axillary, a reduced cyme of 1–2-flowers; bracts 3, foliaceous, narrowly lanceolate to lanceolate-ovate, 4.0–5.5(–7.0) × 1.2–1.8(–2.2) cm, greenish to yellow-green, often with maroon maculation; apex long-acuminate, base attenuate; margin serrate, mainly toward the apex; midrib prominent; indumentum sericeous to villous; outer bracts usually concealing the inner bracts and calyx; pedicels 4–10 mm long, with reddish glands below the floral tube. ***Calyx*** lobes subequal to unequal, narrowly lanceolate, 2.7–3.5 × 0.2–0.5 cm, light green to cream-green, becoming whitish distally, densely covered with long, hyaline, uniseriate eglandular trichomes, forming a sericeous-villous indumentum, margins subentire. ***Corolla*** 6.0–6.5 cm long, 16–17 mm at the widest point (apex), bilabiate, slightly curved, narrow at the base and gradually expanding toward the mid-upper portion, not ventricose; lower lobe narrowly ovate-triangular, patent, 14–15 mm long, 5–6 mm wide at base, densely covered with capitate-glandular trichomes, with a dark maroon-purple basal blotch; lateral and upper lobes fused into a hood; lateral lobes completely reflexed, triangular to semi-ovate, ca. 20 mm long; upper lobes fused, 8–10 mm wide, 9–10 mm long, apex bilobed, each lobe shortly acute to rounded; outer surface densely covered with long, hyaline, uniseriate, eglandular trichomes, forming a sericeous-villous indumentum, inner surface pilose and with glandular trichomes, white to cream-white, with two dark purple longitudinal bands parallel to the axis of the corolla tube, originating near the dorsal lobe and extending for 28–31 mm, frequently converging toward the base of the lower lip. ***Stamens*** 4; filaments 5.9–6.0 cm long, adnate to the base of the corolla tube for ca. 1 mm long, white, glabrous proximally, distally bearing transparent uniseriate trichomes and capitate-glandular indumentum; anthers ca. 3 × 2 mm, subquadrate, included within the corolla tube; stamens exserted during the male phase of anthesis. Nectary consisting of a single, irregularly lobed dorsal gland, glabrous, 3.0–3.2 mm high. ***Ovary*** 5–5.3 × 3.5–4.0 mm, densely tomentose, with transparent uniseriate trichomes; style reddish, proximally with transparent uniseriate trichomes, distally sparsely bearing capitate-glandular trichomes; stigma stomatomorphic, papillose, included within the corolla tube. ***Fruits*** oblong to ovoid, ca. 1.4 × 1.0 cm, densely hirsute; seeds ellipsoid, brown, ca. 1.5–1.8 mm long.

#### Phenology.

According to the herbarium specimens studied, they were collected with flowers in the months of August, October, November and January, and with fruits in November..

#### Etymology.

The specific epithet puipuiense refers to the Bosque de Protección Pui Pui, located in the central rainforest of Peru.

#### Distribution and preliminary assessment of conservation status.

*Columnea puipuiense* occurs in the Bosque de Protección Pui Pui, Junin Department, Peru, where it inhabits primary montane forest on rocky slopes. The substrate is characterized by a thin humus layer overlying semi-decomposed organic matter. The understory is dominated by herbaceous ferns, with associated species of Rubiaceae, Melastomataceae, and Poaceae (*Chusquea* spp.), as well as slender lianas. Trees are densely covered with mosses and epiphytes. Cyatheaceae (*Cyathea* spp.), Cunoniaceae (*Weinmannia* spp.), and Alzateaceae (*Alzatea verticillata* Ruiz & Pav.) are particularly abundant; the latter is characterized by a fenestrate trunk, smooth, often reddish-brown bark, and a scaly rhytidome (Fig. 4).

*Columnea puipuiense* has also be found in secondary montane forest within the buffer zone of protected natural areas in the Pasco Department, an area considered highly vulnerable due to habitat loss and forest fragmentation. *Columnea puipuiense* is assessed as Near Threatened (NT) following IUCN guidelines (2024). The species has a wide geographic range, with an Extent of Occurrence (EOO) of 113,148 km^2^ and an Area of Occupancy (AOO) of 12,000 km^2^ calculated using a 2 × 2 km grid. These values are well above the thresholds for a threatened category under IUCN Criterion B. However, despite its broad distribution, the effective Area of Occupancy is smaller than the calculated AOO due to habitat loss and fragmentation caused by agricultural expansion, road construction, and selective logging, particularly outside protected areas. This is supported by spatially explicit analyses of forest loss that reveal persistent hotspots of high to extremely high deforestation in the central Peruvian forest (Huánuco-Pasco-Junín), overlapping buffer zones of protected areas such as the Parque Nacional Yanachaga Chemillén and the Bosque de Protección Pui Pui (MINAM 2026), indicating ongoing habitat degradation and fragmentation. As a result, a continuing decline in habitat quality is inferred, highlighting the importance of continued habitat monitoring.

#### Comments.

*Columnea puipuiense* is placed within *Columnea*, and is closely allied to *Columnea albiflora*, *C. medicinalis* and *C. tessmannii* based on its epiphytic habit, strongly anisophyllous leaves, and elongate tubular corolla. However, it is distinguished by a combination of vegetative and floral characters.

Vegetatively, *Columnea puipuiense* is characterized by an abaxial leaf surface that is olive green and distinctly discolorous, with maroon-red coloration concentrated toward the apex and margins (Fig. 2A). This contrasts with the usually uniformly pale yellowish green abaxial surface with reddish primary and secondary veins in *C. albiflora*, and a uniformly violet to purple abaxial leaf surface in *C. tessmannii*.

Floral morphology provides additional distinguishing characters. The corolla *Columnea puipuiense* is consistently white to cream-white (Fig. 2D–F), differing from the yellow corolla of *C. tessmannii* and from the variable coloration (white, cream, or yellow with orange lobes) observed in *C. medicinalis*. Also, the corolla indumentum is distinctive, being densely distributed throughout the entire length of the tube and composed of long, hyaline, uniseriate, eglandular trichomes, forming a uniform sericeous-villous indumentum (Fig. 2D–F). In contrast, *C. albiflora* exhibits a sericeous indumentum, whereas in *C. tessmannii* the indumentum is sericeous-villous but restricted to the upper portion of the corolla.

The morphology of the corolla lobes also clearly separates the species. *Columnea puipuiense* has corolla lateral lobes reflexed (Fig. 2F), in contrast to the spreading lobes observed in *C. albiflora*, *C. tessmannii*, and *C. medicinalis*. In addition, the corolla ventral lobe is narrowly ovate-triangular, patent, and cream-white (Fig. 2D, E), differing from the ovate and reflexed lobe in *C. albiflora*, the ovate-lanceolate and reflexed lobe in *C. tessmannii*, and the lanceolate and reflexed lobe in *C. medicinalis*.

In summary, *Columnea puipuiense* is distinguished by a combination of morphological characters, among which the most notable area the corolla color, white to cream-white; the outer surface of the corolla tube, densely covered with long, hyaline, uniseriate, and eglandular trichomes, forming a sericeous-villous indumentum (Fig. 2D–F); and the corolla lateral lobes completely reflexed (Fig. 2F). It also has an olive-green distinctly discolorous abaxial leaf surface, frequently with maroon-red apices and margins, as well as additional spots of the same color, especially toward the apex and margins (Fig. 2A). The diagnostic characters distinguishing *Columnea puipuiense* from its similar species are detailed in Table 1.

**Table 1.** Comparison of *Columnea puipuiense* with morphological similar species.

Character	*Columnea puipuiense*	*Columnea albiflora*	*Columnea tessmannii*	*Columnea medicinalis*
**Leaf blade shape**	Elliptic-lanceolate to oblanceolate	Oblanceolate to falcate	Slightly falcate	Elliptic
**Abaxial leaf surface**	Olive-green, distinctly discolorous, with maroon-red coloration at the apex and margins	Pale green, usually uniform	Generally violet to purple	Green with reddish-brown tones
**Calyx lobes**	Narrowly lanceolate, light green to cream-green	Lanceolate	Narrowly lanceolate	Lanceolate
**Corolla length**	5.8–6 cm	5–7 cm	ca. 6.1 cm	5.0–6.6 cm
**Corolla indumentum**	Densely covered throughout its entire length with long, hyaline, uniseriate eglandular trichomes (sericeous-villous)	Sericeous externally	Sericeous-villous, mostly apical	Sericeous, internally glabrous
**Corolla color**	White to cream-white	White	Yellowish to greenish	White to cream
**Lateral corolla lobes**	Reflexed	Spreading	Semi-ovate, patent	Triangular
**Ventral corolla lobe**	Narrowly ovate-triangular	Ovate	Ovate-lanceolate	Lanceolate

#### Additional specimen examined.

Peru • Pasco: Prov. Oxapampa, Dist. Chontabamba, carretera Chontabamba a la Suiza, bosque secundario montano zona de amortiguamiento, 10°33'42"S, 75°27'23"W, 2100 m, 11 Nov. 2004 (fl.), *A. Monteagudo, A. Peña, R. Francis. C. Arias & C. Rojas 7645* (AMAZ, HOXA!, HUT, MO, MOL-F, USM!). • Prov. Oxapampa, Dist. Chontabamba, fundo Muller, bosque secundario sobre suelo rocoso, 10°35'17"S, 75°27'54"W, 2294 m, 25 nov. 2019 (fl., fr.), *L. Valenzuela & A. Peña 13969* (HOXA!). • Prov. Oxapampa, Dist. Chontabamba, Ulcumano Lodge, bosque primario parcialmente intervenido, 10°38'16.812"S, 75°26'4.819"W, 2232 m, 7 Oct. 2025 (fl.), *R. Vásquez, R. Rojas, E. Pinche & J. Flores 51093* (HOXA!, MO!, USM!).

### Columnea valenzuelai

Taxon classification

Plantae

LamialesGesneriaceae

R.Rojas & Vásquez sp. nov.

09580349-2011-583E-B134-16F0DAE3F295

urn:lsid:ipni.org:names:77380395-1

Fig. 3

#### Type.

Peru • Junín: Prov. Concepción, Dist. Andamarca, Bosque de Protección Pui Pui, bosque montano alto, suelo arenoso con abundante materia orgánica con turba. 11°18'11.6"S, 74°58'57.0"W, 3332 m, 26 Sep. 2021 (fl.), *L. Valenzuela, E. Pinche, A. García & A. Pariachi 39996* (holotype: HOXA! [HOXA079063]; isotypes: MO, USM!, MOL-F!).

**Figure 3.**
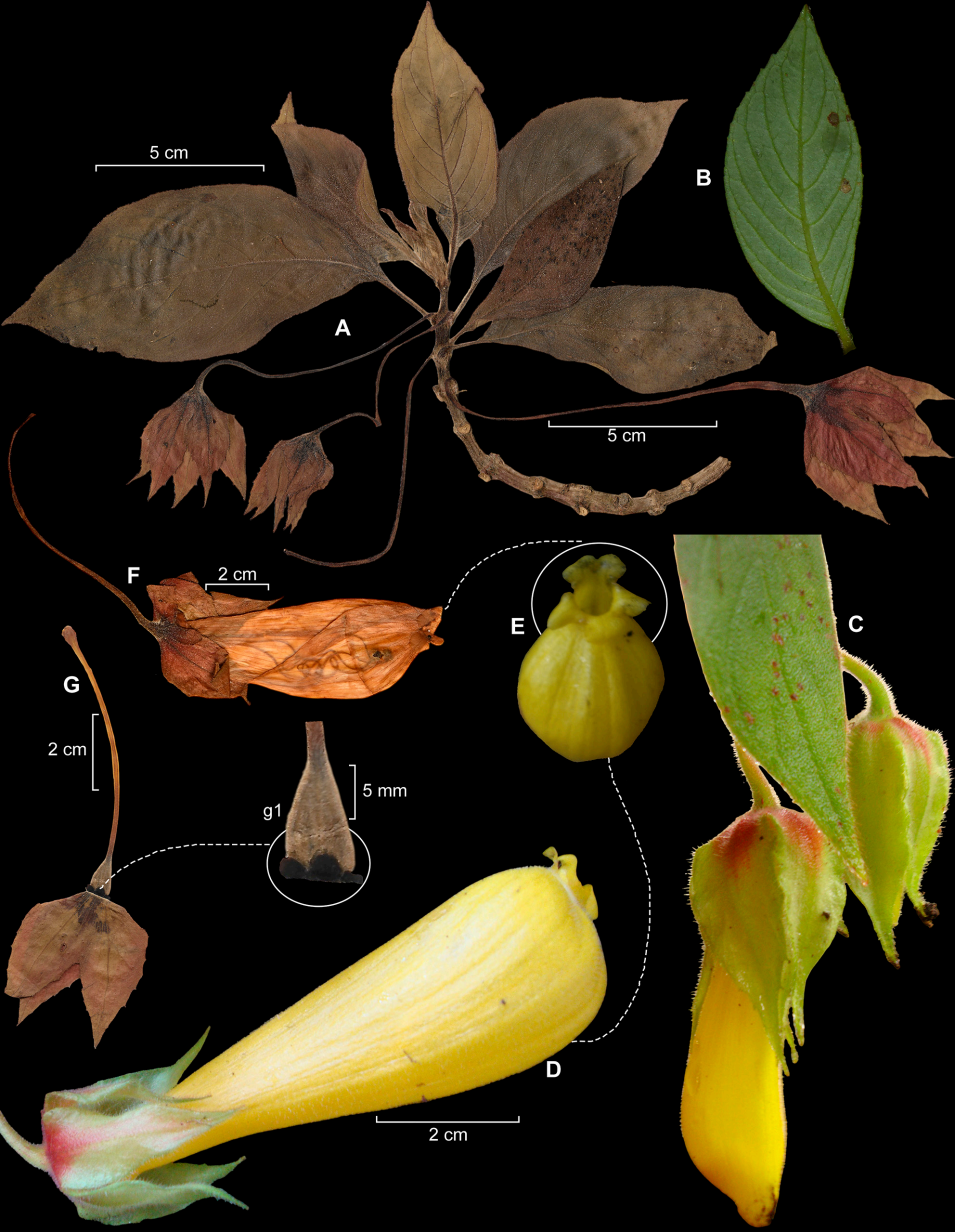
*Columnea valenzuelai* R.Rojas & Vásquez. **A**. Habit; **B**. Abaxial leaf surface; **C**. Immature floral bud; **D**. Corolla; **E**. Corolla lobes; **F**. Lateral corolla portion; **G**. Style and stigma: ovary showing the nectary (**g1**) (**A, B**, **F, G, g1** from L. Valenzuela et al. 39996; **C–E** from L. Valenzuela et al. 28516). Photos by Luis Valenzuela. Plate prepared by Rocío Rojas (**A**, **F**, **G**, **g1**).

**Figure 4.**
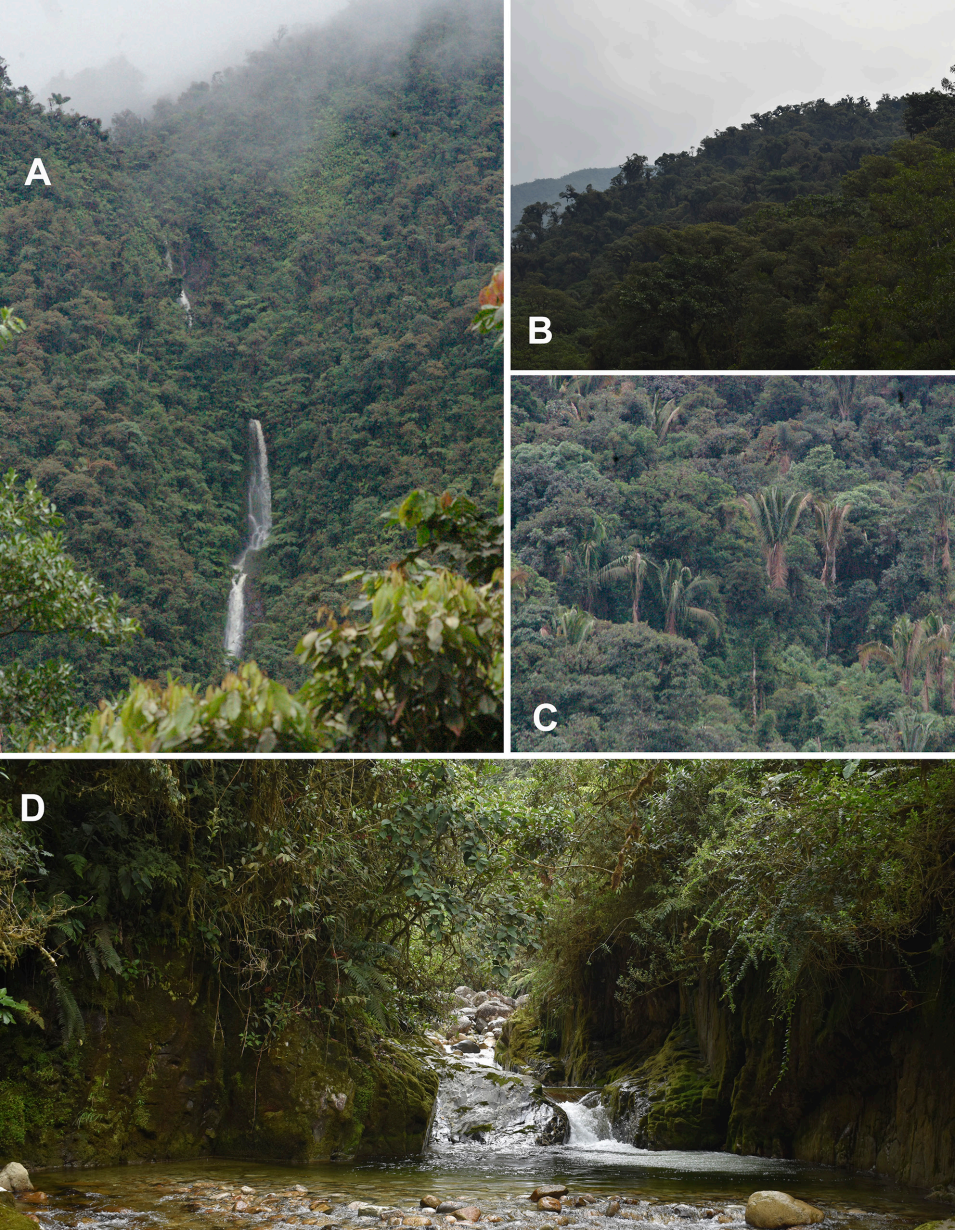
Landscapes of the Bosque de Protección Pui Pui. **A**. Waterfall at Valle Encantado, 1.5 km from marker 03; **B**. Antay valley sector; **C**. *Welfia alfredii* A.J. Hend. & Villalba (Arecaceae); **D**. Rocky cliffs in the Antay Valley sector. **A–D** Photos by Rodolfo Vásquez.

#### Diagnosis.

*Columnea valenzuelai* differs from *Columnea strigosa* Benth. and *Columnea oblongifolia* Rusby; however, it is distinguished by corollas 8.5–9.5 cm long, corolla uniformly yellow with a yellow limb (vs. 2.8–9.5 cm long, orange to reddish-orange with a yellow limb in *C. strigosa*; 4.0–6.5 cm long, yellow or purplish-red with a yellow limb in *C. oblongifolia*); pedicels 6.5–13.0 cm long (vs. 1.5–30.0 cm long in *C. strigosa*; 2.9–9.0 cm long in *C. oblongifolia*), a densely tomentose ovary (vs. villous to sericeous in *C. strigosa*; tomentose in *C. oblongifolia*); and a nectary composed of five free glands, with the two dorsal glands enlarged (vs. variable, from five free glands to two dorsal glands connate in *C. strigosa*; consistently with two dorsal glands connate in *C. oblongifolia*).

#### Description.

Suffrutescent epiphytic or terrestrial subshrub, with scandent to ascending shoots. ***Stems*** to 3 m tall, 4.6–5.1 mm in diam., terete, proximally smooth, and glabrescent, distally pilose; internodes 2.0–6.0 cm long, limited to apex of shoots; internodes slightly swollen, leaf scars raised. ***Leaves*** opposite, generally clustered towards the apex of the stem, isophyllous to slightly anisophyllous; blade 1.5–13 × 1.7–5.5 cm, ovate to elliptic, apex acute to brevi-acuminate, base cuneate to obtuse, slightly oblique, adaxially green, appressed pilose to hirsute, abaxially olive green, appressed pilose to hirsute; both sides with uniseriate transparent trichomes, sericeous on veins with uniseriate hairs; lateral veins 6–8 per side; margin denticulate, margin vesture red-purple with uniseriate trichomes to glandular hairs; petioles 1.0–2.5 cm long, greenish to reddish-yellow, sericeous with transparent uniseriate trichomes to glandular hairs. ***Inflorescences*** reduced to 1 axillary flower. ***Flowers*** subtended by 1 bract, occasionally caducous, ca. 6 × 2 mm, linear, green, densely hirsute. ***Pedicels*** 6.5–13 cm long, pendent in the axils, sericeous with transparent uniseriate trichomes or bearing reddish glandular. ***Calyx*** clasping the corolla base; lobes equal to subequal, 30–46 × 15–21 mm, ovate to broadly ovate, apex acuminate, greenish, reddish centrally, externally with glandular trichomes; margin subentire to briefly denticulate (distally with 4–6 teeth per side). ***Corolla*** 8.5–9.5 cm long, 3.0–4.5 cm wide at widest point, 6–10(–12) mm wide before limb, 8–16 mm wide at base, tubular, ventricose, constricted at base and before limb, uniformly yellow with yellow limb; 2 dorsal lobes shortly fused, 6–6.5 × 5.5–6(–7) mm, exterior surface pilose with uniseriate transparent trichomes, interior often with glandular trichomes internally and dorsally; lateral lobes 5–6 × 5–6(–7) mm, semiorbicular, slightly reflexed; ventral lobe 5–5.5 × 5–6 mm, semiorbicular, slightly reflexed; short denticulated margin to premorse. ***Filaments*** 8.5–9.3 cm long, adnate to base of corolla tube for 1–2 mm, white, glabrous; anthers 3 mm long, 3 mm wide, subquadrate, included in corolla tube. Nectary of 5 free glands, two dorsal enlarged. ***Ovary*** ca. 10 mm long, 4–5 mm wide, densely tomentose, with uniseriate transparent trichomes; style white, with uniseriate transparent trichomes proximally and sparsely with capitate-glandular trichomes distally; stigma bilobed, papillose, included in corolla tube. ***Fruits*** not seen.

#### Phenology.

According to the examined herbarium specimens, flowering occurs in September and October.

#### Etymology.

The specific epithet valenzuelai honors Luis Valenzuela Gamarra, a highly active taxonomist and field botanist with strong specialization in Orchidaceae and the Andean–Amazonian flora, and a key contributor to the scientific knowledge of the central Peruvian rainforest. He actively works on the study of floristic diversity in protected natural areas of the central rainforest and has an extensive scientific output in taxonomy, ecology, and conservation.

#### Distribution and preliminary assessment of conservation status.

One of the ecoregions within the Bosque de Protección Pui Pui is the Peruvian Yungas ecoregion, corresponding to the montane forest, commonly known as the cloud forest or high jungle, which constitutes the Amazonian portion of the landscape. Its altitudinal range extends from 1400 to 3500 meters above sea level. *Columnea valenzuelai* was found at an altitude of 3300 to 3350 meters above sea level. Above 3000 meters, this cloud forest floristic composition changes markedly, characterized by Asteraceae and Melastomataceae as the predominant families, followed by Ericaceae and Myrsinaceae (SERNANP 2019). *Columnea valenzuelai* is assessed as Critically Endangered (CR) under criteria B1ab(iii) following IUCN guidelines (2024), is known only from a locality within the Bosque de Protección Pui Pui, central Peru. It has an extremely restricted geographic range, with an Extent of Occurrence (EOO) of 0.305 km^2^ and an Area of Occupancy (AOO) of 8 km^2^, calculated using a 2 × 2 km. Given its narrow distribution and the ongoing pressures affecting the habitat within and around the protected area, as supported by documented forest loss within the Bosque de Protección Pui Pui totaling 314 ha between 2001 and 2024, with persistent annual loss and recent values ranging from 6 to 16 ha/year (MINAM 2026), as well as by spatial analyses identifying persistent hotspots of deforestation in the central Peruvian forest (Huánuco-Pasco-Junín), which may directly affect habitat integrity, a continuing decline in the extent and quality of habitat is inferred (UICN subcriterion b(iii)). *Columnea valenzuelai* inhabits humid premontane to montane forest within the Bosque de Protección Pui Pui. It grows as a climbing or scandent plant, occurring in shaded understory and along forest margins.

#### Comments.

*Columnea valenzuelai* is morphologically similar to *Columnea strigosa* and *Columnea oblongifolia*, sharing a suffrutescent habit, often epiphytic or terrestrial, with elongate, scandent to ascending shoots, opposite, leaves clustered toward the stem apex, and a tubular, ventricose corolla. However, it is readily distinguished by a consistent set of floral and vegetative characters. Among the three species *Columnea valenzuelai* is unique in its uniformly yellow corolla 8.5–9.5 cm long (Fig. 3D), whereas *C. strigosa* displays orange to reddish-orange corollas with a yellow limb and marked variation in size (2.8–9.5 cm long), and *C. oblongifolia* has shorter corollas (4.0–6.5 cm long) that are yellow or red-purple with a yellow limb and typically more clearly bilabiate. The *Columnea valenzuelai* corolla is tubular and strongly ventricose (Fig. 3E), with semiorbicular lobes, contrasting with the more variable and often more deeply bilabiate corollas of *C. strigosa* and *C. oblongifolia*.

Additionally, the nectary structure provides a key diagnostic feature: *Columnea valenzuelai* shows five free glands with two dorsal glands enlarged (Fig. 3G, g1), whereas *C. strigosa* exhibits a variable nectary, ranging from five free glands to two connate dorsal glands, and *C. oblongifolia* consistently presents two connate dorsal glands. This character is stable in *Columnea valenzuelai* and supports its recognition as a distinct species.

In summary, the combination of corolla size and coloration, together with nectary structure, clearly distinguishes *Columnea valenzuelai* from *C. strigosa* and *C. oblongifolia*. The diagnostic characters distinguishing *Columnea valenzuelai* from its related species are compared in Table 2.

**Table 2.** Comparison of *Columnea valenzuelai* with morphological similar species.

Character	*Columnea valenzuelai*	*Columnea strigosa*	*Columnea oblongifolia*
**Leaf shape**	Ovate to elliptic	Ovate to elliptic, rarely large ovate-orbicular	Oblong to elliptic
**Number of flowers per inflorescence**	1	1	1
**Pedicel length**	6.5–13.0 cm	1.5–30.0 cm (highly variable)	2.9–9.0 cm
**Calyx lobes shape**	ovate to broadly ovate, acuminate	Ovate to lanceolate	Linear to lanceolate
**Calyx lobe size**	30–46 × 15–21 mm	5–40 × 3–12 mm	10–23 × 1–3 mm
**Calyx lobes margin**	Subentire to briefly denticulate (distally with 4–6 teeth per side)	Serrate (at least basally)	Entire
**Corolla color**	Uniformly yellow with yellow limb	Orange, rarely reddish orange with yellow limb	Yellow or red purple with yellow limb
**Corolla length**	8.5–9.5 cm	2.8–9.5 cm	4.0–6.5 cm
**Dorsal corolla lobes**	Not forming a bilabiate structure	Fused, forming a prominent galea	Fused, forming a less conspicuous galea
**Nectary**	Constant: 5 free glands with the 2 dorsal enlarged	Variable: 5 free glands, 2 connate dorsal	Constant: 2 connate dorsal glands
**Ovary indumentum**	Densely tomentose	Villous to sericeous	Tomentose

Ecologically, *Columnea valenzuelai* occupies montane forest habitats similar to *C. strigosa* and *C. oblongifolia*. In Perú, *Columnea valenzuelai* is restricted to the Bosque de Protección Pui Pui (Junín Department), where it has been recorded in humid montane forests, suggesting a high degree of endemism and a limited geographic range. In contrast, *C. strigosa* has a broader distribution in the country, occurring primarily in the Amazonas and Cajamarca Departments, with additional records from Cusco, where it inhabits humid montane and cloud forest across a wide elevational gradient. *C. oblongifolia*, in turn, is distributed in southern Perú, particularly in the Cusco Department, where it occurs in Andean cloud forests.

#### Additional specimen examined.

Peru • Junín: Prov. Concepción, Dist. Andamarca, Bosque de Protección Pui Pui, bosque montano alto suelo arenoso cubierto con abundante turba, 11°18'11.2"S, 74°58'53.7"W, 3305 m, 20 Sep. 2021 (fl.), *L. Valenzuela, A. García, E. Pinche & R. Portocarrero 39908* (HOXA!). • Prov. Satipo, Dist. Pampa Hermosa, comunidad campesina Santa Rosa de Toldopampa, caserío Jatun Talhuis, Bosque de Protección Pui Pui, bosque puna “estepa espinosa”, 11°21'09"S, 74°56'12"W, 3309 m, 13 Oct. 2014 (fl.), *L. Valenzuela, J. Flores & G. Shareba 28516* (HOXA!, MO, USM).

## Supplementary Material

XML Treatment for Columnea puipuiense

XML Treatment for Columnea valenzuelai

## Additional information

### Artificial Intelligence (AI) use

The authors accept full responsibility for the content of the manuscript, including the disclosure of any use of AI.

No AI tools were used in the preparation of this manuscript.

### Author ORCIDs

R.delP. Rojas Gonzáles https://orcid.org/0000-0001-9494-1036

R. Vásquez Martínez https://orcid.org/0000-0002-2282-5009

E. Pinche Shareva https://orcid.org/0009-0002-7432-3657

### Data availability

All of the data that support the findings of this study are available in the main text.
